# Reengineering the clinical research enterprise to involve more community clinicians

**DOI:** 10.1186/1748-5908-6-36

**Published:** 2011-04-04

**Authors:** Gery Ryan, Claude Berrebi, Megan Beckett, Stephanie Taylor, Elaine Quiter, Michelle Cho, Harold Pincus, Katherine Kahn

**Affiliations:** 1 RAND Health, Santa Monica, CA, USA; 2 RAND-University of Pittsburgh Health Institute, Pittsburgh, PA, USA; 3 Department of Psychiatry, Columbia University, New York, NY, USA; 4 New York-Presbyterian Hospital, New York, NY, USA; 5 David Geffen School of Medicine at UCLA, Department of Medicine, Los Angeles, CA, USA; 6 US Department of Veterans Affairs, Health Services Research and Development Office, VA Greater Los Angeles Healthcare System, Los Angeles, CA, USA; 7 UCLA School of Public Health, Community Health Sciences, Los Angeles, CA, USA; 8 Compass Lexecon, Oakland, CA, USA

## Abstract

**Background:**

The National Institutes of Health has called for expansion of practice-based research to improve the clinical research enterprise.

**Methods:**

This paper presents a model for the reorganization of clinical research to foster long-term participation by community clinicians.

Based on the literature and interviews with clinicians and other stakeholders, we posited a model, conducted further interviews to test the viability of the model, and further adapted it.

**Results:**

We propose a three-dimensional system of checks and balances to support community clinicians using research support organizations, community outreach, a web-based registry of clinicians and studies, web-based training services, quality audits, and a feedback mechanism for clinicians engaged in research.

**Conclusions:**

The proposed model is designed to offer a systemic mechanism to address current barriers that prevent clinicians from participation in research. Transparent mechanisms to guarantee the safety of patients and the integrity of the research enterprise paired with efficiencies and economies of scale are maintained by centralizing some of the functions. Assigning other responsibilities to more local levels assures flexibility with respect to the size of the clinician networks and the changing needs of researchers.

## Introduction

"The scale and complexity of today's biomedical research problems increasingly demand that scientists move beyond the confines of their own discipline and explore new organizational models for team science [1]." With this statement, Dr. Elias Zerhouni galvanized the research community to consider how best to "reengineer the clinical research enterprise" and "develop new partnerships among organized patient communities, community-based clinicians, and academic researchers." In two previous articles, we identified specific barriers community clinicians face in participating in clinical research and laid out specific strategies that will facilitate participation of these clinicians and their associated healthcare organizations in clinical research [2,3]. However, these strategies alone are insufficient for recruiting and supporting enough community-based clinicians to "reengineer" the clinical research enterprise. Rather, more systematic changes to the infrastructure of the research enterprise will be required [4].

The current system relies heavily on two methods for recruiting and supporting clinicians in research. The traditional method relies on principal investigators (PIs) and research teams to recruit clinicians into studies [5]. The second, newer system involves collaborative networks of clinicians and healthcare communities interested in research [6,7]. Both strategies have had some success, but not nearly enough to populate the current-or future-multiple, large-scale clinical studies needed to test the efficacy of a new generation of biomedical and genetically engineered interventions [4,8]. For both of these methods, recruiting clinicians and patients for each new study is time-intensive, expensive, and inefficient [9,10]. Further, clinicians who agree to participate in these studies often feel overly burdened and poorly reimbursed, provide data of variable quality, and are reluctant to participate in future work [10,11]. Although clinical trial networks (CTNs) and practice-based research networks (PBRNs), for example [12], have been successful in engaging clinicians in multiple studies over time, CTNs typically involve collaborations of small groups of clinicians (*e.g.*, 50 to 200 for most CTNs), whereas PBRNs are not in a position to provide sufficient infrastructural support to consistently engage clinicians over time [1]. To meet the dual goals of recruiting and retaining large numbers of community clinicians in research over a period of time, this paper proposes an alternative for reengineering the research system. This paper describes the components and interrelationships of such a system, the advantages the system would offer, the requirements for such a system to work, and expected measurable outcomes.

## Methods

We were funded by the National Institutes of Health (NIH) as a component of the roadmap to help develop a conceptual model for a system that would allow a large number of clinicians to participate in clinical research while they care for patients in their office settings. To accomplish this goal, we conducted a classic formative evaluation [13,14]. Formative evaluations help develop and improve programs from an early stage by (a) identifying the size and scope of key issues, (b) generating and selecting among potential solutions, and (c) adapting existing interventions to fit cultural and organizational cultures. To this end, we first identified the main problems with and potential solutions to the current research system via a review of the literature and in-depth interviews with community and academic clinicians and other key stakeholders in the current research enterprise. For our literature reviews, we relied on both keyword searches as well as suggestions from those people we interviewed, where we focused primarily on clinical trials, PBRNs, and quality improvement collaboratives (*e.g.*, the Institute for Healthcare Improvement) [15]. We began to develop our initial model only after we reached a point where additional interviews and searches were revealing few new ideas. We then refined the model and confirmed its feasibility by interviewing additional clinicians and stakeholders, as well as returning to interview some of those who we had spoken with previously. Details of the methodology are described in companion manuscripts [2,3].

The RAND Institutional Review Board reviewed these materials and procedures prior to the start of the interviews.

## Results

### A three-dimensional approach to supporting the involvement of community clinicians in clinical research

**Figure 1:**
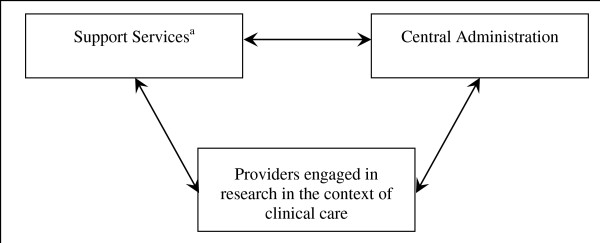
**The three dimensions of a reengineered research enterprise**. ^a^The set of support services include research support organizations (RSOs), community outreach, a web-based registry of providers and studies (ROPS), web-based training services, quality audits, and a feedback mechanism for clinicians engaged in research in the context of clinical care.

Our research suggests that any approach that attempts to engage and maintain large numbers of community clinicians in the research enterprise will need to take into account three dimensions (Figure 1). First, for any approach to work, it must result in the creation of a large, diverse, and stable network of clinicians who will participate in both clinical research and clinical care [2,3]. Second, to assist this network, a well-integrated set of services must be established to identify, recruit, retain, and support those clinicians in a manner consistent with the standards that characterize high-quality research. Third, to ensure consistency and quality across the network, an administrative and coordinating capacity will be needed to oversee the functions of the network and its provision of services. Table 1 contrasts the tasks and responsibilities of the current system with our proposed three-dimensional research system. For example, in the current system, ongoing support for clinicians and staff collecting the data is varied and dependent on available resources and the capacity of PIs, CTNs, and PBRNs. In the proposed approach, ongoing support is more consistent across studies and research sites and delivered by well-supported and incentivized research support organizations (RSOs).

**Table 1:** Research tasks and responsibilities in the current system and the proposed reengineered research enterprise

Key Research Tasks	Current System	Proposed Approach
Research design	PIs	PIs
Protocol development	PIs	PIs, with input from clinician panel
Recruitment of clinicians	PIs recruit for single studies; CTNs and PBRNs recruit for multiple studies but are often discipline- and disease-specific	ROPS acts as the source for a large pool of studies; RSOs recruit for multiple studies across disciplines and diseases
Training		
General research training	Usually incorporated into study-specific training by PIs	Web-based training
Study-specific training	PIs	PIs, with option to link to web-based training
Ongoing support for clinicians and staff collecting data	Varied and dependent on available resources and capacity by PIs, CTNs, PBRNs	More consistent across studies and research sites and delivered by well-supported and incentivized RSOs
Quality assurance	PIs	Quality assurance at the site level; PIs for data
Clinician feedback	Via PIs	Via PIs, ROPS, RSOs, and protocol review panels
Use of study results	PIs via direct communication and peer-reviewed articles	ROPS to entire relevant network, not just to those who participated in the study; plus PIs via peer-reviewed articles

PI = principal investigator; CTN = clinical trial network; PBRN = practice-based research network; ROPS = registry of providers and services; RSO = research support organization.

These three dimensions should work together as a vehicle to further the efficiency and productivity of research; create networks that are diverse and representative of all healthcare and research stakeholders by engaging significantly more managed care, fee-for-service, and safety-net providers from diverse communities; and promote a wide variety of studies applicable to large numbers of patients. In addition, this three-dimensional approach offers a structure for organizing incentives and administering finances associated with research conducted in clinical settings. It can be characterized as a flexible tool to be shared across all funders of clinical research (potentially including, *e.g.*, the NIH or individual institutes within it, pharmaceutical companies, specialty societies, and even the RSOs themselves).

### A network of clinicians

Approximately 3% of physicians in the United States serve as investigators [16], and these are typically in association with academic health centers. Although no accurate source for the total number of community physicians involved in research could be identified, the Federation of Practice-Based Research Networks' Inventory of Networks lists approximately 9,750 physicians (1% to 2% of American physicians) as members [17].

Recruiting and maintaining a stable network of community clinicians would reduce the time and effort that have been needed to recruit and train such clinicians each time a study is begun. A stable network of community clinicians, including specialists in family medicine, pediatrics, and internal medicine as well as other community clinicians (*e.g.*, obstetric/gynecologist physicians, dentists, and nurse practitioners), and subspecialists would gain experience across time and facilitate the conduct of diverse studies with diverse patient participants [4,8]. A large, diverse, experienced set of clinicians would accommodate implementation of many study designs, both sequentially and simultaneously.

### An integrated set of services for supporting participating clinicians

In the current system, the kinds and amounts of support given to clinicians vary across studies and are determined primarily by the resources available to PIs, CTNs, and PBRNs. Variation in resources and support across studies makes it difficult for community physicians to estimate whether they can afford to participate in research on an ongoing basis. To motivate a stable cadre of clinicians to integrate research into their practices, a variety of support services will need to be developed and implemented predictably for all clinicians, regardless of specialty or previous experience.

### Research support organizations

To recruit, support, and retain clinicians and to overcome the innumerable barriers and disincentives that clinicians face in participating in clinical research, RSOs will be needed to directly interface with and support the unique needs of clinicians and their clinical settings as they attempt to conduct clinical research [3]. These organizations could be formed by academic health centers, academic research organizations, PBRNs, clinical research organizations, CTNs, multispecialty groups, health management organizations (HMOs), community hospitals, or clinical practices. They could evolve from the newly funded NIH-sponsored Clinical Translational Science Awards (CTSAs). Based on RAND's estimations of network size and our assumption that RSOs will probably be most responsive if they limit their services to no more than 1,500 clinicians at a time, we estimate that approximately 30 to 40 RSOs will be needed to cover the full range of geographic, ethnic, and medical diversity in the country while achieving appropriate economies of scale (see below).

RSOs will develop a systems approach based upon their collective experience and knowledge but will work locally with individual clinicians to recruit clinicians to (a) participate in a stable research program, assist them in the registration process, help match them with appropriate studies, and address any initial clinician questions and concerns; (b) work with individual clinicians to reorganize office infrastructure to facilitate research, identify and train appropriate research staff, or assist in patient recruitment efforts; (c) provide easily accessible support to help reduce the day-to-day burden of problem solving for office staff; and (d) take responsibility for encouraging clinicians to remain in the network after completing their first study [3].

One of the research support organizations' key roles will be to help reduce the day-to-day problem solving and research burden that typically falls to the office staff. This can be done through a range of mechanisms, including employment of an adequate number of professional research coordinators, trainers, and problem solvers and the provision of online and printed resources for clinicians and staff. RSOs also will need to recognize the critical role that personal and trusted relationships play in establishing and maintaining network participation.

RSOs will need to be reimbursed for their efforts by a central administrative function (see below). Ideally, RSOs will be incentivized to assist community clinicians to engage in research via market principles of competition. Clinicians, who would be free to change RSOs at will, would gravitate toward those RSOs that provide the best combination of services relative to requirements. The central administrative function and its more hands-on affiliates (RSOs) will need to be flexible about the specific strategies used to support clinicians so RSOs can be free to work innovatively with clinicians to achieve their goals. To maintain quality and uniformity in the services provided and the clinicians recruited, RSOs will need to engage with the administration and adhere to established standards for retaining membership.

However, even when RSOs are available to provide guidance and support, five additional types of services will be required to help initiate and sustain community clinician engagement: community outreach, a registry of providers and studies (ROPS), web-based training services, quality audits, and a clinician feedback mechanism.

### Community outreach

Currently, most community outreach efforts are study-specific, with few resources dedicated to fostering broader, more long-term relationships between community clinicians and the research enterprise. Long-term outreach would engage community members and clinicians by providing research-related opportunities and information, reducing the burden to participate in research, and soliciting advice and feedback about the research process. Clinicians, for example, would be updated about research opportunities available to them and their patients and reminded of the importance of clinical research to clinicians and their professional organizations and the value patients derive from research conducted in community settings. A strong community outreach approach would also foster greater clinician and community participation in the design and implementation of the research itself. Seeking clinician and community engagement and feedback would help ensure that study designs and logistics were more likely to take into consideration the realities and constraints of community practices and that communities would have a greater voice in identifying the research questions and topics that were most relevant to them. This suggestion is consistent with the RE-AIM (research, effectiveness, adoption, implementation, maintenance) framework, one of several evaluation frameworks developed to support the assessment of interventions in terms of the translatability and public health impact of health promotion [18-20].

### Registry of providers and studies

Once providers are informed of a new initiative to recruit them to long-term research participation, they will need to be able to register their interests and learn about salient study opportunities. A web-based ROPS could provide this service, listing clinicians' names, specialties, interests, and research experience in terms of completed training, studies initiated, and patients enrolled. Although some clinician recruitment is population-based, with attention paid to long-term clinician involvement [21], much is done in an *ad hoc *manner, with little attention paid to systematically monitoring or encouraging clinician involvement across multiple studies.

### Web-based training services

To ensure that clinicians and key office staff have basic research skills, web-based training, allowing clinicians and staff to participate at their convenience without leaving their clinical practices, should be provided and its completion documented in clinicians' registry profiles. Although some study-specific training and quality assurance procedures would be needed, a standardized approach to these activities across studies, sites, clinicians, and staff should provide substantial economies of scale and opportunities for improved research outcomes. Currently, basic research training is often incorporated into study-specific training, increasing the burden on clinicians and staff who engage in multiple studies.

### Quality audits

To ensure that research environments foster high quality research, RSOs might institute and oversee a quality assurance program that will conduct routine audits within the clinicians' offices. These efforts will assure clinicians, as well as other stakeholders, that research conducted in their offices is of the highest quality.

### Clinician feedback mechanism

Currently, clinicians who participate in clinical trials have little voice in designing those studies and even less influence on the research enterprise as a whole [21]. When clinicians have concerns about protocols, enrollment, data, and/or reimbursement, they should be able to communicate those concerns through the registry and/or with their RSOs. At the national level, the registry would collect concerns and issues identified by clinicians and their staff. These concerns would be aggregated and passed on to both PIs as well as the appropriate NIH funding sources. At the local level, RSOs would be responsible for collecting and responding to such clinicians' concerns. Since RSOs are also responsible for motivating clinicians to participate and supporting them, they have a vested interest in listening and responding to clinician concerns. In this way, clinicians will be assured that their voices are heard, particularly with respect to quality and safety concerns, throughout the research process.

### An administrative and coordinating center

To support the network of clinicians and deliver the six categories of services described above, a central structure with coordinating capacity will be required. This central structure would administer the web-based registries and training programs; oversee the quality assurance process; select, manage, and remunerate the core group of RSOs; and facilitate feedback mechanisms among PIs, RSOs, and clinicians. The key role of the administrative personnel would be to take the lead in making this three-dimensional structure, and its potential impact on the clinical research enterprise, a reality.

### Advantages of a three-dimensional system

Establishing a diverse representation of clinicians, patients, and treatment settings for a wide array of studies that will be applicable to a large segment of the patient population, with results available for review and dissemination by community clinicians as well as by academic researchers (thus providing a deeper understanding of the underlying biology and promoting interdisciplinary research teams), is ambitious. Yet a well-coordinated program would enhance the overall quality and efficiency of the clinical research enterprise by introducing transparency and accountability to enhance safeguards for data quality and engender greater trust among clinicians and the public [22]. Having a stable network of clinicians would, in the long run (*e.g.*, 10 or more years), lead to efficiencies in patient recruitment, data collection, and data transfer, as well as to a more stable pool of researchers and patients who are readily engaged in clinical research. If implemented carefully, the visibility of this reengineered research enterprise should lead to increases in research participation by clinicians and patients and increases in public trust in clinical research. Ultimately, such increases are likely to promote uses of clinical research findings into communities and facilitate the delivery of better care.

Using a three-dimensional approach also provides the potential for checks and balances across the three dimensions that will allow safe, efficient, and effective conduct of research by community clinicians. Each of the three proposed dimensions can implement procedures or controls to ensure that high-quality data collection standards are met and human subjects are protected. The central administrative structure could take responsibility for maintaining the registry, conducting quality audits, and providing mechanisms for clinician feedback. RSOs could be responsible for coordinating services associated with recruitment and clinician support and could also consult with those responsible for the registry and quality assurance in deciding on the appropriate and measurable indicators to use in assessing study-specific and across-study performance. Properly aligned incentives will reward a research support organization's effort for recruiting and sustaining large numbers of clinicians in high quality research. Finally, clinicians could indicate their satisfaction with an RSO via the clinician feedback mechanism and through their actual rate of participation with clinical research. If clinicians "vote with their feet" by not enrolling patients or by disengaging in the research enterprise, that information could be fed back to ROPS and, ultimately, to the central administration.

The administrative and financial implications of this proposed system are nontrivial. Although there are some administrative and financial costs needed to oversee the national program (including the development and maintenance of the registry), most of the resources will be allocated to local RSOs. The RSOs will receive the most administrative and financial resources because they are the most critical and proximate component for removing burdens and barriers from community clinicians and their staff. Although the CTSA has made strides in this direction, much of their community engagement has been on a study-by-study basis. In contrast, our approach advocates for a program that will establish a stable national network of local community practices. The network of community practices would have the ability to engage in a wide range of research opportunities and would be funded by the various institutes of the NIH that wished to have access to such a network.

We would expect that the various institutes of the NIH would fund the national infrastructure and contribute significantly to the initial development of RSOs. Over time, however, we would expect that the federal contribution to RSOs would diminish. RSOs stand to gain in terms of prestige and/or profit if they are able to successfully develop and maintain a large and diverse research network of community providers. Since consortiums of organizations will have to compete with each other to become RSOs and RSOs will compete with each other to attract studies funded by either the NIH or the pharmaceutical industry, we would anticipate that RSOs would contribute at least some resources (directly or in kind) to the development and maintenance of their research network. As RSOs become more efficient, they should be able to recover a higher portion of their infrastructural costs directly from studies and thus begin to move toward self-sufficiency.

### How might we measure the success or failure of such a system?

Several measures might be used to gauge the success of this effort (Table 2). For example, after implementing the new system, we might expect to see improvements in the number, diversity, and representativeness of patients involved in clinical research, as well as improvement in the kinds of direct and indirect care that such patients receive in practices engaged in research. Likewise, we would expect to see more clinicians with greater diversity engaged in clinical research, with less turnover and greater satisfaction with the research process. If such a system were designed and implemented as suggested, we would expect to see the number and diversity of patients, clinicians, and RSOs involved in clinical research increase over time. The scope, number, and size of studies should also increase, and more studies should be completed on time and on budget. If the quality control mechanisms are properly enabled, we should also expect higher quality data and increases in patient, clinician, and RSO satisfaction and public trust. While some of these outcomes are easy to quantify because they are consistent with explicitly stated goals, others are more conceptual, unanticipated, and only likely to translate into highly valued results with time.

**Table 2:** Potential measures for evaluating the success or failure of a reengineered research enterprise relative to its major stakeholders

Patients	Clinicians	Research support organizations (RSOs)	NIH and the biomedical research enterprise
a) Number, diversity, and representativeness of patients involved with CR	a) Number, diversity, and representativeness of clinicians and settings involved with CR	a) Number of organizations engaged in the support of CR	a) Efficiency of the CR process (*e.g.*, measured as the number of studies completed as planned on schedule)
b) Safety of patients involved with CR	b) Number of studies and types of studies in which clinicians participate	b) Number of studies and types of studies in which RSOs participate	b) Number of studies being conducted
c) Retention throughout the tenure of the research study; participation rates in long-term outcome studies	c) Degree of engagement of clinicians with components of CR	c) Degree of engagement of RSOs with components of CR	c) Distribution of study types being conducted
d) Improved care as a direct consequence of CR participation	d) Efficiency of participation with CR	d) Efficiency of participation with CR	d) Number and proportion of patients who sustain an adverse outcome
e) Improved care as an indirect effect of CR	e) Clinician bankruptcy as a consequence of involvement with CR	e) RSO dropout from research and financial difficulties following participation in CR	e) Number and proportion of patients who are subjects of Institutional Review Board infractions
f) Patient satisfaction and trust with CR	f) Clinician satisfaction with CR	f) RSO satisfaction with CR	f) Effect of biomedical research findings on the practice of medicine
g) Outcomes associated with the conduct of CR	g) Stability of clinician as a participant in CR	g) Stability of RSOs as a participant in CR	g) Effect of biomedical research findings on the health of the people
	h) Repeat participation of clinicians in CR		h) Long-term CR capabilities
			i) Degree of public trust

## Discussion

We have proposed a general design for a large, well-supported, stable system to increase the participation of community clinicians in clinical research that includes a set of checks and balances between a network of clinicians, a set of service providers, and a small administrative structure. The proposed design specifically recognizes that any serious effort to involve large numbers of community clinicians in clinical research must address the current barriers that prevent more clinicians from participation and must include transparent mechanisms to guarantee the safety of patients and the integrity of the research enterprise. Efficiencies and economies of scale are maintained by centralizing some of the functions, while assigning other responsibilities to more local levels. The system is also designed to be scalable in terms of the size of the clinician network and adaptable to the changing needs of researchers. Thus, the system might be adapted for use as a single national program or across a region, a state, or a large group of academic and/or managed care affiliates.

The rationale for developing this program is the simple notion that engaging more community clinicians in the research enterprise will enhance the nation's research capacity and improve the quality of care over time. It represents a long-term investment in the research enterprise, with success depending upon leadership, commitment, and support from stakeholders already engaged and invested in research. If we want to engage community clinicians in research on a large scale, can we afford not to do this?

## Competing interests

The authors declare that they have no competing interests.

## Authors' contributions

GR, MB, and KK designed the study and drafted the manuscript. EQ, CB, ST, MC, and HP guided study design and read and revised the manuscript. All authors read and approved the final manuscript.
